# Editors’ pick: transcriptomes of 1000 genomes

**DOI:** 10.1186/2041-2223-4-17

**Published:** 2013-10-18

**Authors:** Antti Sajantila

**Affiliations:** 1 Department of Forensic Medicine, Hjelt Institute, University of Helsinki, Helsinki, Finland; 2 Institute of Applied Genetics, Department of Forensic and Investigative Genetics, University of North, Texas Health Science Center, Fort Worth, TX, USA

## 

Next generation sequencing (NGS) technologies facilitate massive human DNA sequence variation data to be produced in a remarkable manner and speed. The functional effects of these variants can now also be analyzed by high-throughput RNA sequencing (RNA-seq) of the transcriptome. Prior to RNA-seq, analysis of human gene expression was performed by expression arrays with more limited coverage.

In the recent issue of *Nature*, Lappalainen et al*.*[1] have, for the first time, used the full capacity of RNA-seq to create the largest existing catalog of potential causative functional variants in the genome and to characterize transcriptome variation in human populations in a subset of individuals from the 1000 Genomes Project. They reported a deep analysis of high-quality mRNA and miRNA sequences from lymphoblast cell lines from > 450 individuals belonging to five populations (CEPH-CEU Europeans, Finns, British, Toscani Italians, and Yoruba from Nigeria).

The authors addressed several questions with their dataset. First, they investigated human transcriptome variation at the population level, which can manifest in overall expression levels or in splicing (in one gene). In a genome-wide perspective, Lappalainen et al. showed that population differences account for about 3% of the total variation, but they also identified 263–4.379 genes with differential expression and/or transcript ratios between population pairs. In the pairwise analysis, they observed something interesting: the African-European population pairs had a higher contribution of genes with differential splicing (75 to 85%) than the European populations had between each other (6 to 40%). Although this phenomenon has not been previously observed in humans, it is in agreement with phylogenetic differences between species being better captured by splicing rather than expression levels.

Lappalainen et al*.* analyzed 644 autosomal miRNAs that could be quantified from > 50% of the individuals. Sixty of those miRNAs (9.3%) had significant *cis*-expression QTLs (eQTLs) for miRNA expression levels, indicating that genetic effects on miRNA expression are more widespread than had been known. The authors also provide evidence for the existence of feedback loops for mRNA and miRNA genes to have an effect on each other’s expression supporting the idea that miRNAs offer robustness in the expression programs.

Altogether, Lappalainen et al. have produced the largest and most diverse catalogue of *cis*-regulatory variants in a single tissue to date. The authors used their data to analyze the functional properties of the newly found set of regulatory variants and transcriptome effects of protein-truncating loss-of-function variants. This collaborative effort further used the potential of RNA-seq to discover regulatory variants that affect not only expression levels but also splicing. Of the 7.825 genes with expression QTLs (eQTLs), 34% have a second, independent eQTL for any of their exons. The authors concluded that in the transcriptome there exists allelic heterogeneity for regulatory effects on a single gene and independence of exons within the same gene.

As an ancillary benefit, the authors demonstrated that RNA-seq data is consistent even when produced in different laboratories, with more detailed analysis in a companion paper in *Nature Biotechnology*[2]. Namely, in the replicate analyses of RNA-seq data from seven laboratories, there was a smaller amount of variation among the laboratories than seen among the individuals.

The study is a great example of integration of genomic sequencing and cellular phenotype data. Obviously, the amount of data produced in this study is enormous, and it can easily be expected that the data will also be used to support other pivotal work. Meanwhile, it will take some time to completely digest the magnitude of data and appreciate the impact it will have on functional genomics.

## Competing interests

The authors declare that they have no competing interests.
